# How pre-pregnancy body mass index influences health-related quality of life during pregnancy: a serial multiple mediation analysis

**DOI:** 10.1186/s12884-025-08564-2

**Published:** 2025-12-09

**Authors:** Hyun Ji Park, Hyunjeong Shin, Won-Oak Oh, Songi Jeon, Inhae Cho

**Affiliations:** 1 Korea University College of Nursing, 145 Anam-ro, Sungbuk-gu, Seoul, 02841 Korea; 2 Department of Nursing, Catholic Kwandong University, Gangneung, Korea; 3 Inje University College of Nursing, Busan, Korea

**Keywords:** Body mass index, Quality of life, Pregnant women, Depression, Sleep, Health perception, Serial multiple mediation analysis, Path model

## Abstract

**Background:**

Recent studies have suggested pre-pregnancy body weight plays a role in women’s health-related quality of life (HRQOL) during pregnancy. However, the mechanisms underlying this relationship remain unclear. This study aims to explore the associations between pre-pregnancy body mass index (BMI) and HRQOL during pregnancy, focusing on the potential mediating roles of depressive symptoms, sleep disturbances, and general health perception.

**Methods:**

A cross-sectional correlational study was conducted with 240 pregnant Korean women using self-report questionnaires. A multi-mediation path model was developed to assess the interrelationships among variables. Path analysis was performed using AMOS 26.0, and serial multiple mediation analysis with phantom variables was used to evaluate the mediating roles of sleep disturbances, depressive symptoms, and health perception.

**Results:**

The proposed path model demonstrated a good fit, explaining 56.8% of the variance in pregnant women’s HRQOL. Pre-pregnancy BMI showed relationships with HRQOL indirectly through sleep disturbances, depressive symptoms, and general health perception, indicating no direct association between pre-pregnancy BMI and HRQOL during pregnancy. Among the hypothesized paths, however, the path of sleep disturbance → HRQOL and the sequential path of pre-pregnancy BMI → sleep disturbance → HRQOL were not significant. These findings suggest that although sleep disturbance does influence HRQOL, its effect is largely mediated through depressive symptoms and general health perception.

**Conclusions:**

To promote HRQOL during pregnancy, healthcare professionals should educate women of reproductive age on the importance of maintaining a healthy BMI before conception. Additionally, prenatal care should incorporate simultaneous screening for sleep disturbances and depressive symptoms, particularly among women with elevated pre-pregnancy BMI.

## Background

Overweight and obesity are important public health challenges worldwide, with profound impact on individual health and quality of life (QOL) [1]. Recent studies have demonstrated a relationship between obesity and QOL in pregnant women [2]. Especially, pre-pregnancy body weight plays a role in women’s health-related quality of life (HRQOL) during pregnancy [2–5]. A systematic review indicated that women who were overweight or obese before pregnancy tend to be at increased risk of obstetric complications such as gestational diabetes mellitus (GDM), preeclampsia, stillbirth, and hypertension [2, 6]. Women who were overweight before pregnancy exhibited lower vitality compared to those with a normal weight [5], suggesting an indirect impact on their health perception and overall HRQOL. Poor general well-being due to pregnancy-related complications may further deteriorate women’s HRQOL [3]. Given these risks, it could be expected that maternal obesity may be negatively related to QOL during pregnancy [7]. However, research investigating the specific mechanisms through which pre-pregnancy body weight is linked to QOL in this population remains limited [7].

Tang et al. [4] reported that a higher body mass index (BMI) is associated with poorer sleep during pregnancy in a prospective cohort study. Another study also reported that women who were overweight or obese before pregnancy tend to experience poor sleep [2]. Literature explains that increased fat deposition around the upper airway in obese women may lead obstruction of breathing and it increases risk of sleep apnea and other sleep disorders [4, 8]. Insufficient sleep negatively affects daytime vitality, thereby influencing individual health perceptions and HRQOL [9]. A recent systematic review found a strong association between poor sleep and lower HRQOL during pregnancy [10]. Thus, we hypothesized that:

- H1: Higher pre-pregnancy BMI is associated with increased sleep disturbance.
- H2: Increased sleep disturbance is associated with lower HRQOL.
- H3: Increased sleep disturbance is associated with poorer health perception.
- H4: Better health perception is associated with higher HRQOL.

Sleep disturbances are common among pregnant women and result in decreased sleep quality [11]. Studies indicate that 59–80% of pregnant women experience sleep disturbances, which tend to worsen as pregnancy progresses [4]. Poor sleep plays an important role in perinatal mood disorders. A systematic review found that poor sleep quality is associated with increased depressive symptoms during pregnancy [12]. Given the high prevalence of perinatal depression and its extensive effects on both mothers and infants [13], depressive symptoms are closely related to negative health perception and lower HRQOL [14]. A European cohort study reported that pregnant women with more depressive symptoms exhibited lower health perception and all domains of QOL, as the strongest predictor of QOL [15]. Thus, we hypothesized that:

- H5: Increased sleep disturbance is associated with more severe depressive symptoms.
- H6: More severe depressive symptoms are associated with poorer health perception.
- H4: Better health perception is associated with higher HRQOL.
- H7: More severe depressive symptoms are associated with lower HRQOL.

Taken together, previous studies suggest that sleep disturbances, depressive symptoms, and general health perception may mediate the relationship between pre-pregnancy BMI and HRQOL in pregnant women. To date, however, these variables have not been examined simultaneously. Understanding these mechanisms is essential for predicting and improving HRQOL in pregnant women with overweight or obesity.

### Study purpose

**Fig. 1:**
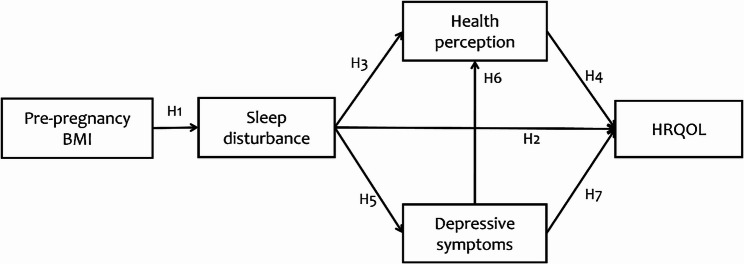
Hypothetical multi-mediating path model. Note. BMI = body mass index; HRQOL = health-related quality of life

This study aims to explore the associations between pre-pregnancy BMI and HRQOL during pregnancy, focusing on the potential mediating roles of depressive symptoms, sleep disturbances, and general health perception. To examine interrelationships among the variables, we constructed a hypothetical multi-mediating path model (Fig. 1).

## Methods

### Participants and data collection procedure

**Fig. 2:**
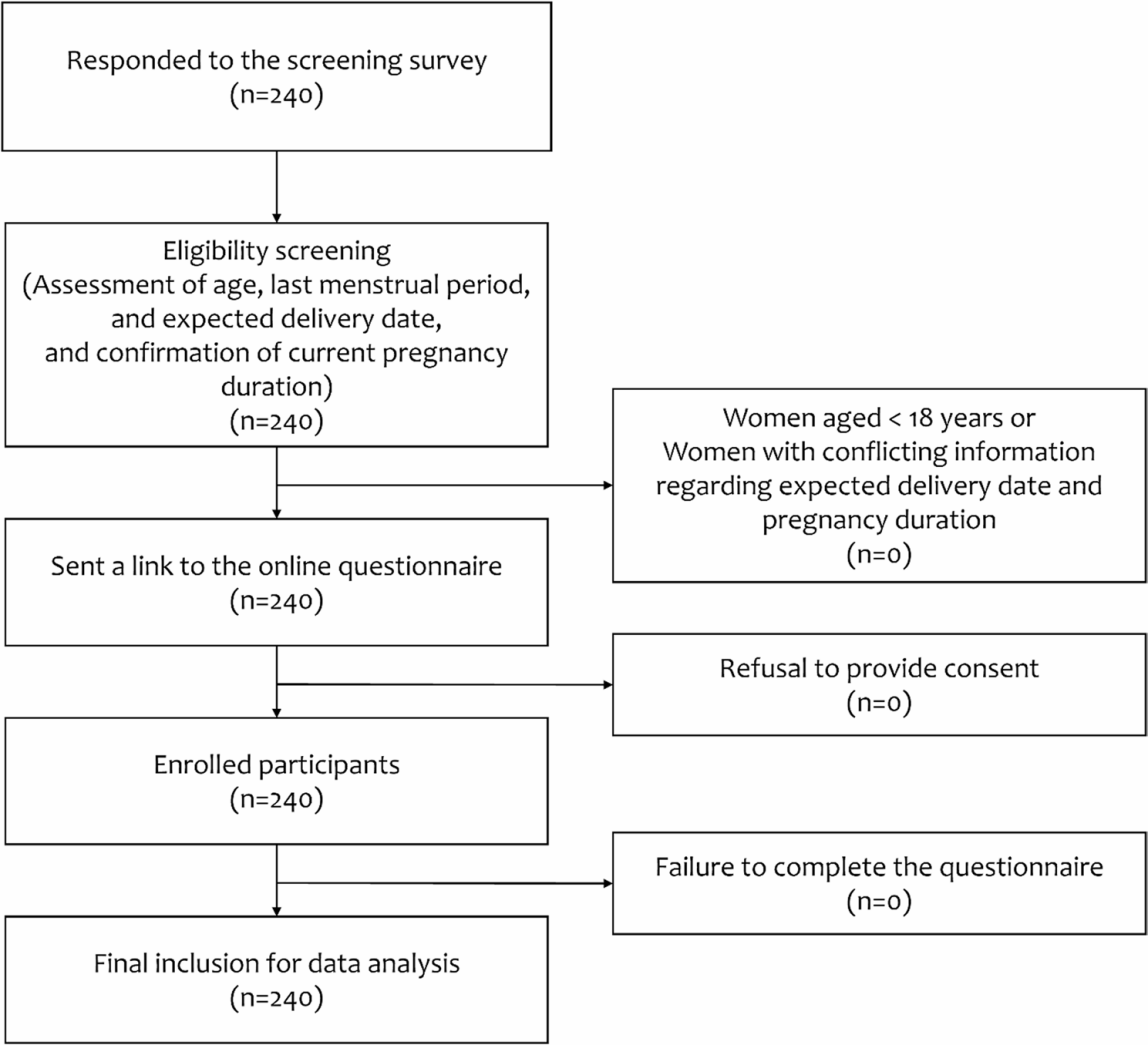
Flow diagram of participant recruitment

This is a cross-sectional study. Data were collected between November 2020 and May 2021. Participants were recruited through convenience sampling from online communities focused on pregnancy and childbirths in South Korea. The eligibility criteria for participation were as follows: (a) aged ≥ 18 years, (b) pregnant at the time of the survey, and (c) able to read and understand Korean. To facilitate recruitment, an announcement about the study was posted on an online community bulletin board. Women interested in participating were asked to provide details such as their last menstrual period, current pregnancy duration, expected delivery date, and mobile phone number to receive a link to the online questionnaire. Upon confirmation of pregnancy, the survey URL was sent to them. A total of 240 pregnant women responded to the screening survey, all of whom completed the self-reported online questionnaire. The process of participant recruitment is shown in Fig. 2.

A priori power analysis was conducted using G*Power software (version 3.1.9.4), applying F tests within the framework of linear multiple regression with a fixed model and R² deviation from zero. The minimum required sample size was calculated to be 129 participants, assuming a statistical power of 0.95, an alpha level of 0.05, and a medium effect size (f² = 0.15). Additionally, a minimum of 200 cases is recommended for path analysis using maximum likelihood estimation [16]. Considering these criteria, the final sample size of 240 participants was deemed sufficient to ensure adequate statistical sensitivity for detecting relationships among the study variables.

### Measures

#### HRQOL

 HRQOL was measured using the Korean version of the World Health Organization Quality of Life Assessment Instrument [17]. The scale includes two items measuring overall quality of life and general health, seven items assessing physical health, six items evaluating psychological health, three items examining social relationships, and eight items measuring environmental health. Responses are scored on a 5-point Likert-type scale, with higher scores indicating better HRQOL. The Cronbach’s alpha coefficient for this study was 0.95.

#### Sleep disturbance

 Sleep disturbance levels were measured using the Korean version of the Sleep Quality Index [18], derived from the Pittsburgh Sleep Quality Index [19]. For this study, the original Korean version was modified by removing items related to sleep medication use and questions that required responses from someone the participant shared a bed with. The modified scale comprised one item assessing overall sleep quality and nine items measuring sleep disturbances. Responses are recorded on a 4-point Likert-type scale (0 = no problem at all, 3 = severe difficulty), with higher scores indicating poorer sleep quality and more severe sleep disturbances. The Cronbach’s alpha coefficient of the original Korean version was 0.84 [18], and that of the modified scale used in the present study was 0.76.

#### Depressive symptoms

 Depressive symptoms were assessed using the Korean version of the Center for Epidemiologic Studies-Depression Scale [20]. This scale comprises 20 items rated on a 4-point Likert-type scale (0 = none of the time, 3 = all of the time), reflecting the frequency of depressive symptoms experienced in the past week. Higher scores indicate more severe depressive symptoms. The Cronbach’s alpha coefficient was 0.93 in the present study.

#### Health perception 

Health perception was measured using a single global item asking pregnant women to rate their general health on a 5-point Likert-type scale (1 = poor, 5 = excellent).

### Data analysis

Descriptive statistics and correlation coefficients for study variables were computed using SPSS for Windows (version 25.0). No data was missing. Normality of study variables was verified using skewness (-3 < desired value < 3) and kurtosis (-10 < desired value < 10), confirming normal distributions (skewness: -0.52 ~ 2.19; kurtosis: -0.52 ~ 8.15) [16].

Path model testing was performed using the AMOS software (version 26.0). Prior to testing model fit, tolerance (desired value > 0.10) and variance inflation factor (desired value < 10) were calculated to examine multi-collinearity problems. Model fit was evaluated using the following goodness-of-fit indices: chi-square value, goodness of fit index (GFI; desired value > 0.90), adjusted goodness of fit index (AGFI; desired value > 0.90), Tucker-Lewis index (TLI; desired value > 0.95), comparative fit index (CFI; desired value > 0.95), root mean square error of approximation (RMSEA; desired value < 0.08), and standardized root mean square residual (SRMR: desired value < 0.08) [16].

To evaluate the multi-mediation effects of sleep disturbance, depressive symptoms, and health perception between pre-pregnancy BMI and HRQOL, a serial multiple mediation analysis using phantom variables was conducted.

## Results

### Participant characteristics

The participants had a mean age of 32.85 years (*SD* = 3.43, range: 25–45 years). Over half (57.1%) were employed, and the majority (93.3%) had completed at least a college education. The average pregnancy duration was 23.20 weeks (*SD* = 8.59, range: 4–38 weeks), with 41.2% in the second trimester. Most participants (94.2%) reported no diagnosed pregnancy complications. Approximately three-quarters of the participants had a normal BMI before pregnancy (73.8%) and had no prior birth experience (72.5%). Table 1 presents the demographic and obstetric characteristics of the participants.

**Table 1:** Characteristics of the participants (*n* = 240)

Variables	*n* (%)	Mean ± SD	Range
Age (years)		32.85 ± 3.43	25–45
Employment			
Employed	137 (57.1)		
Unemployed	103 (42.9)		
Monthly household income			
Less than 1,000,000 won	6 (2.5)		
1,000,000 ~ 3,000,000 won	70 (29.2)		
3,000,000 ~ 5,000,000 won	100 (41.7)		
More than 5,000,000 won	64 (26.7)		
Education			
High school or less	16 (6.7)		
College	188 (78.3)		
Graduate school	36 (15.0)		
Pregnancy duration (weeks)		23.20 ± 8.59	4–38
1st trimester	47 (19.6)		
2nd trimester	100 (41.6)		
3rd trimester	93 (38.8)		
Parity			
Yes	66 (27.5)		
No	174 (72.5)		
Pregnancy complications			
Yes	14 (5.8)		
No	226 (94.2)		
Pre-pregnancy BMI		21.18 ± 3.07	15.00–35.40
< 18.5	40 (16.7)		
18.5 ≤ BMI < 25	177 (73.8)		
≥ 25	23 (9.6)		

^†^ The average Korean household income (monthly) in 2021 was 4,640,000 won (Statistics Korea)

*BMI* Body mass index

### Relationships among study variables

HRQOL was significantly correlated with depressive symptoms (*r* = -0.69, *p* < 0.01), sleep disturbance (*r* = -0.37, *p* < 0.01), health perception (*r* = 0.62, *p* < 0.01), and gestational trimesters (*r* = 0.14, *p* = 0.03). Table 2 provides descriptive statistics for the study variables and their correlation coefficients. 

**Table 2:** Descriptive statistics and correlations among study variables (*n* = 240)

Variables	Possible range	Mean	SD	1	2	3	4	5	6
1. Pre-pregnancy BMI	–	21.18	3.07	1.00					
2. Depressive symptoms	0–60	17.69	10.74	0.02	1.00				
3. Sleep disturbance	0–30	13.72	5.40	0.16*	0.40**	1.00			
4. Health perception	1–5	3.44	0.80	-0.05	-0.52**	-0.31**	1.00		
5. Gestational trimesters	1–3	2.19	0.74	0.07	-0.13*	0.01	0.21**	1.00	
6. HRQOL	26–130	85.40	16.93	-0.01	-0.69**	-0.37**	0.62**	0.14*	1.00

*BMI* Body mass index, *HRQOL* Health-related quality of life

* *p* < 0.05, ** *p* < 0.01

### Fitness of the path model

Before testing the model fit, tolerance values and the variance inflation factor were calculated to examine multicollinearity, confirming there were no multi-collinearity problems among the study variables (tolerance 0.52 ~ 0.94, variance inflation factor 1.07 ~ 1.93).

As shown in Table 2, statistically significant relationships were found between gestational trimesters and depressive symptoms, health perception, and HRQOL. In order to more accurately estimate the direct and indirect effects of the study variables on women’s HRQOL and to enhance the reliability and stability of the model, we included “gestational trimesters” as a covariate in the path model analysis.

Evaluation of the hypothetical path model showed that the model fit was good (*χ2* = 2.55 [4, 240], *p* = 0.64, GFI = 0.99, AGFI = 0.98, TLI = 0.99, CFI = 0.99, RMSEA = 0.01, SRMR = 0.02). Among the seven hypothesized paths in the model, one (sleep disturbance → HRQOL) was not statistically significant. Modification indices were examined during the model evaluation process; however, no substantial indices were suggested by AMOS. This indicates that the initially specified model was statistically adequate and did not require post hoc modifications.

### Direct and indirect effects of study variables on HRQOL

**Fig. 3:**
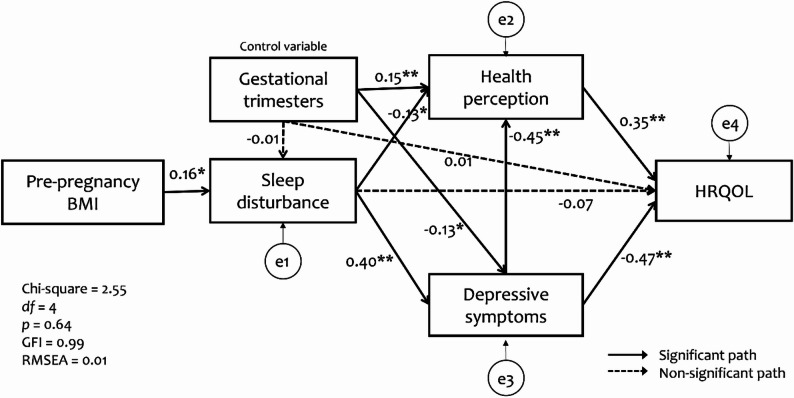
Results of the multi-mediation path model testing. Note. * *p* < 0.05, **p < 0.01; BMI = body mass index; HRQOL = health-related quality of life; GFI = goodness-of-fit index; RMSEA = root mean square error of approximation

Table 3 presents the direct, (summative) indirect, and total effects of each predictive variable on HRQOL. Depressive symptoms (β = -0.47, *p* < 0.01) and health perception (β = 0.35, *p* < 0.01) had significant direct effects on HRQOL. However, the direct effects of sleep disturbance (β = -0.07, *p* = 0.11) and gestational trimesters (β = 0.01, *p* = 0.99) were not statistically significant. Gestational trimesters (β = 0.13, *p =* 0.02) had only indirect positive effects on HRQOL. Collectively, four predictive variables (pre-pregnancy BMI, depressive symptoms, sleep disturbance, and health perception) accounted for 56.8% of the variance in pregnant women’s HRQOL (Fig. 3).

**Table 3:** The standardized effect coefficients of the study variables on HRQOL in the path model (*n* = 240)

Predicting variables	Direct effects	(Summative) Indirect effects	Total effects
Depressive symptoms	-0.47**	-0.16**	-0.63**
Sleep disturbance	-0.07	-0.30**	-0.37**
Health perception	0.35**		0.35**
Pre-pregnancy BMI		-0.06**	-0.06**

*BMI* Body mass index, *HRQOL* Health-related quality of life

** *p* < 0.01

### Mediating effects of sleep Disturbance, depressive symptoms, and health perception

Although the summative indirect effect of pre-pregnancy BMI on HRQOL was significant in Table 3, the individual indirect effect was different according to the detailed path. In the multi-mediation model, pre-pregnancy BMI influenced HRQOL through three pathways: through sleep disturbance and health perception (b = -0.04, *p* < 0.05); through sleep disturbance and depressive symptoms (b = -0.17, *p* < 0.01); and through sleep disturbance, depressive symptoms, and health perception (b = -0.04, *p* < 0.01). However, the path of pre-pregnancy BMI → sleep disturbance → HRQOL (b = -0.07, *p* = 0.08) was not significant (Table 4).

**Table 4:** Regression coefficients in the serial multiple mediation analysis (*n* = 240)

Paths	b	SE	β
Pre-pregnancy BMI → sleep disturbance → HRQOL	-0.07	0.05	-0.01
Pre-pregnancy BMI → sleep disturbance → health perception → HRQOL	-0.04*	0.03	-0.04
Pre-pregnancy BMI → sleep disturbance → depressive symptoms → HRQOL	-0.17*	0.08	-0.16
Pre-pregnancy BMI → sleep disturbance → depressive symptoms → health perception → HRQOL	-0.06**	0.03	-0.05
Sleep disturbance → health perception → HRQOL	-0.04*	0.07	-0.14
Sleep disturbance → depressive symptoms → HRQOL	-0.17**	0.11	-0.58
Sleep disturbance → depressive symptoms → health perception → HRQOL	-0.06**	0.05	-0.20
Depressive symptoms → health perception → HRQOL	-0.06**	0.05	-0.25

*BMI* Body mass index, *HRQOL* Health-related quality of life, *b* unstandardized coefficients, *β* standardized coefficients, *SE* Standard error

* *p* < 0.05; ** *p* < 0.01

## Discussion

This study developed and tested a hypothetical multi-mediation path model to explore the associations between pre-pregnancy BMI and HRQOL during pregnancy, focusing on the potential mediating roles of depressive symptoms, sleep disturbances, and general health perception. The mediation process outlined in the present study shows potential pathways through which these variables are associated with HRQOL.

This study found that the path from pre-pregnancy BMI to HRQOL mediated sequentially by sleep disturbance, depressive symptoms, and general health perception was statistically significant. The findings indicate that pregnant women with lower pre-pregnancy BMI tended to experience fewer sleep disturbances, which were associated with lower levels of depressive symptoms. Pregnant women who feel less depressed might have better health perception, ultimately contributing to better HRQOL. These findings suggest that maintaining a healthy weight prior to conception may promote maternal well-being during pregnancy. Accordingly, healthcare professionals should educate women on the importance of preconception weight management [21].

Consistent with our findings, previous research has reported a negative association between BMI at beginning of pregnancy and the physical health during pregnancy [7]. Another study also reported differences in daytime sleepiness and QOL between obese and non-obese pregnant women, particularly during the third trimester [22]. Our results further suggest that the diminished HRQOL observed in pregnant women who were overweight or obese prior to conception is primarily attributable to the severity of depressive symptoms, which stem from sleep disturbances. Therefore, pre-pregnancy BMI itself may not be directly associated with HRQOL during pregnancy but rather exerts its influence indirectly through sleep and psychosocial pathways. Yang et al. [11] stated that higher early-pregnancy BMI was associated with poorer sleep quality later in pregnancy, and this is partly due to breathing difficulties during sleep. Sleep disturbances accompanied by breathing problems in obese pregnant women have also been linked to an increased risk of adverse pregnancy outcomes, such as preeclampsia [23, 24]. Given their mediating role in HRQOL, sleep and depressive symptoms should be carefully considered when providing care for pregnant women who were overweight or obese prior to conception. It is important to note, however, that our model did not include a direct path from pre-pregnancy BMI to HRQOL. This decision was based on our theoretical assumption that the influence of BMI operates entirely through psychosocial and perceptual mediators. Accordingly, the absence of a direct effect should not be interpreted as a tested null finding, but rather as a structural feature of the model. Future studies may consider testing alternative models that include a direct path to further examine this relationship.

Among the study variables, depressive symptoms emerged as the strongest predictor of HRQOL in our model, suggesting their central role in shaping pregnant women’s HRQOL. This finding indicates that psychological distress may play a more substantial role than physical factors such as BMI, in the observed associations. In the present study, depressive symptoms were directly associated with all study variables except pre-pregnancy BMI. Moreover, depressive symptoms mediated the relationships of pre-pregnancy BMI and sleep disturbance with HRQOL, being associated with HRQOL both directly and indirectly. These results suggest that early identification and management of depressive symptoms should be prioritized in antenatal care. Sleep complaints may serve as early warning signs of psychological distress, and addressing them promptly could help mitigate the downstream effects on HRQOL. However, some women may perceive depressive symptoms as a ‘normal’ aspect of pregnancy and, as a result, may not seek diagnosis or treatment [25]. Given the strong predictive value of depressive symptoms, routine screening and timely intervention should be considered essential components of prenatal health strategies [26].

In addition to examining the direct and summative indirect relationships between study variables, we evaluated the chain mediation effects of sleep disturbances, depressive symptoms, and general health perception in the pathway from pre-pregnancy BMI to HRQOL. Given the high prevalence of sleep disturbances and depressive symptoms among pregnant women [5, 11], addressing both conditions simultaneously may be necessary for improving HRQOL, particularly in women who were overweight or obese before pregnancy.

However, results from the serial mediation analysis indicated that the path of pre-pregnancy BMI → sleep disturbance → HRQOL was not supported. Path model testing revealed that the direct path from sleep disturbance to HRQOL was not significant. Instead, sleep disturbance showed relationships with HRQOL indirectly through depressive symptoms and general health perception. Given that the significant zero-order correlation was observed between sleep disturbance and HRQOL, it is interesting findings. This discrepancy may be attributed to an indirect-only effect, where the influence of sleep disturbance on HRQOL is fully mediated by other variables in the model. The current study found that when depressive symptoms and general health perception were included as mediators in the pathway from sleep disturbance to HRQOL, sleep disturbance no longer had a direct relationship with HRQOL. These findings suggest that although sleep disturbance is associated with HRQOL, it is largely mediated through depressive symptoms and general health perception. Previous studies have reported that sleep has both direct and indirect effects on HRQOL [27–29]; however, some have found that the relationship is not direct, but rather mediated by factors such as depressive symptoms and overall health perception [30, 31]. One possible explanation for the non-significant direct path from sleep disturbance to HRQOL is that the influence of sleep problems may be psychologically internalized before manifesting in perceived QOL [32]. Sleep disturbances often contribute to emotional dysregulation, fatigue, and cognitive impairments, which in turn heighten vulnerability to depressive symptoms. These depressive symptoms may then shape individuals’ subjective evaluations of their health and well-being, thereby influencing HRQOL. In this sense, sleep disturbance may not directly alter QOL perceptions but rather exert its influence through the psychological and perceptual consequences it generates. This pathway aligns with cognitive appraisal theories, which suggest that individuals’ HRQOL is shaped more by their emotional and perceptual interpretations than by objective health conditions alone [32]. Based on previous research, this tendency may be more salient in younger populations, where sleep disturbance influences HRQOL primarily through mediating factors such as depressive symptoms and general health perception [30, 31, 33]. In contrast, studies involving older adults or individuals with chronic conditions have reported both direct and indirect effects of sleep disturbance on HRQOL [28, 34–36]. Younger individuals, being generally healthier than older adults, may not perceive sleep disturbances as significantly detrimental to their HRQOL. In comparison, older adults or those with chronic health issues may be more sensitive to changes in sleep quality due to increased health vulnerabilities. Given these findings, interventions aimed at improving HRQOL among pregnant women experiencing sleep disturbances should not focus solely on sleep-related factors. Instead, a more comprehensive approach that also addresses psychological well-being (particularly depressive symptoms) and enhances general health perception may be necessary. By targeting these mediating factors, it may be possible to more effectively mitigate the negative impact of sleep disturbances on HRQOL during pregnancy.

While this study offers valuable insights into the factors affecting HRQOL during pregnancy, certain limitations should be acknowledged. In this study, only gestational trimester was included as a covariate in the statistical model. Other potential confounding variables such as parity, socioeconomic status, and pregnancy complications were not accounted for in the path analysis. To enhance the accuracy and robustness of future findings, it is recommended that these factors be adjusted for in subsequent studies. Secondly, participants were recruited via online communities. Although South Korea has a high internet penetration rate (100%), with 94.5% of the population using the internet [37], this recruitment strategy may have biased the sample toward individuals who are more educated, of higher socioeconomic status, and more digitally active. This is likely reflected in the fact that 93% of participants in the present study completed at least a college degree. Such sampling bias may limit the generalizability of the findings, and the resulting homogeneity could influence the observed relationships. Third, in the present study, pre-pregnancy BMI was calculated based on self-reported height and weight prior to conception. It may have been influenced by recall bias and social desirability bias, potentially compromising the accuracy of the data. Furthermore, health perception was assessed using a single global item, which is commonly used in health research and helps reduce respondent burden. However, the use of a single-item measure may limit reliability and hinder the ability to fully capture the variable’s associations with other constructs in the model, potentially leading to an underestimation of its influence. Fourth, the study examined variables at a single point in time, preventing causal inferences. Longitudinal research should be conducted to explore causal relationships. Lastly, although pre-pregnancy BMI was found to influence HRQOL through a serial mediation pathway, the effect size was modest (β = -0.06). Therefore, while elevated BMI may contribute to poorer HRQOL indirectly, its impact should be interpreted with caution and in the context of other stronger mediating factors such as sleep disturbances and depressive symptoms.

## Conclusions

This study provides an understanding of how pre-pregnancy BMI is associated with HRQOL during pregnancy through the mediating roles of sleep disturbances, depressive symptoms, and general health perception. The findings suggest that women who were overweight or obese prior to conception may be more likely to experience sleep disturbances and more depressive symptoms, which can lead to negative health perceptions and reduced HRQOL during pregnancy.

To promote HRQOL during pregnancy, healthcare professionals should educate women of reproductive age about the importance of maintaining a healthy BMI before conception. During pregnancy, antenatal care programs should include early screening and management of sleep disturbances, particularly among women with elevated pre-pregnancy BMI, as sleep problems often precede and exacerbate depressive symptoms. Early interventions to improve sleep quality (e.g., sleep hygiene education, behavioral therapy, relaxation techniques) may consequently reduce the risk of depressive symptoms and foster more positive health perceptions. Such tailored, stepwise approaches derived from the identified serial mediation pathways could help mitigate the downstream negative effects on HRQOL. These findings provide practical guidance for healthcare professionals to develop preventive and therapeutic strategies specifically tailored to women with higher pre-pregnancy BMI.

## Data Availability

The data used for the current study are available from the corresponding author on reasonable request.

## Abbreviations

HRQOL: Health-related quality of life
QOL: Quality of life
BMI: Body mass index
GFI: Goodness of fit index
AGFI: Adjusted goodness of fit index
NFI: Normed fit index
CFI: Comparative fit index
RMSEA: Root mean square error of approximation
SRMR: Standardized root mean square residual

## References

[1] Yilmaz AD, Çalik KY, Budak M. The effect of body mass index on maternal and neonatal health in term pregnancies: a cross-sectional study in Turkey. BMC Pregnancy Childb. 2025;25:572. 10.1186/s12884-025-07690-1. 10.1186/s12884-025-07690-1 PMC12076889 40369476

[2] Lagadec N, Steinecker M, Kapassi A, Magnier AM, Chastang J, Robert S, et al. Factors influencing the quality of life of pregnant women: A systematic review. BMC Pregnancy Childb. 2018;18:455. 10.1186/s12884-018-2087-4. 10.1186/s12884-018-2087-4 PMC6251086 30470200

[3] Ferrans CE, Zerwic JJ, Wilbur JE, Larson JL. Conceptual model of health-related quality of life. J Nurs Scholarsh. 2005;37(4):336–42. 10.1111/j.1547-5069.2005.00058.x. 16396406 10.1111/j.1547-5069.2005.00058.x

[4] Tang Y, Dai F, Razali NS, Tagore S, Chern BSM, Tan KH. Sleep quality and BMI in pregnancy - A prospective cohort study. BMC Pregnancy Childb. 2022;22(1):72. 10.1186/s12884-022-04414-7. 10.1186/s12884-022-04414-7 PMC8793200 35086507

[5] Mourady D, Richa S, Karam R, Papazian T, Moussa FH, El Osta N, et al. Associations between quality of life, physical activity, worry, depression and insomnia: A cross-sectional designed study in healthy pregnant women. PLoS ONE. 2017;12(5):e0178181. 10.1371/journal.pone.0178181. 28542529 10.1371/journal.pone.0178181 PMC5439948

[6] Kurnaz D, Karaçam Z. Effects of maternal obesity on maternal and neonatal health: A systematic review and meta-analysis. Adnan Menderes Univ J Health Sci Fac. 2023;7(2):305–30. 10.46237/amusbfd.1224641.

[7] Amador N, Juárez JM, Guízar JM, Linares B. Quality of life in obese pregnant women: a longitudinal study. Am J Obstet Gynecol. 2008;198(2):e2031–5. 10.1016/j.ajog.2007.08.037. 10.1016/j.ajog.2007.08.037 17981249

[8] Venkata C, Venkateshiah SB. Sleep-disordered breathing during pregnancy. J Am Board Fam Med. 2009;22(2):158–68. 10.3122/jabfm.2009.02.080057. 19264939 10.3122/jabfm.2009.02.080057

[9] Nodine PM, Leiferman JA, Cook PF, Matthews E, Hastings-Tolsma M. The impact of physical activity on sleep during pregnancy: A secondary analysis. Clin Mother Child Health. 2016;13(2):e1000245. 10.4172/2090-7214.1000245.

[10] Peters AEJ, Verspeek LB, Nieuwenhuijze M, Harskamp-van Ginkel MW, Meertens RM. The relation between sleep quality during pregnancy and health-related quality of life-a systematic review. J Matern Fetal Neonatal Med. 2023;36(1):2212829. 10.1080/14767058.2023.2212829. 37197986 10.1080/14767058.2023.2212829

[11] Yang JP, Lin RJ, Sun K, Gao LL. Incidence and correlates of insomnia and its impact on health-related quality of life among Chinese pregnant women: A cross-sectional study. J Reprod Infant Psychol. 2023;41(4):391–402. 10.1080/02646838.2021.2020228. 34989304 10.1080/02646838.2021.2020228

[12] González-Mesa E, Cuenca-Marín C, Suarez-Arana M, Tripiana-Serrano B, Ibrahim-Díez N, Gonzalez-Cazorla A, et al. Poor sleep quality is associated with perinatal depression. A systematic review of last decade scientific literature and meta-analysis. J Perinat Med. 2019;47(7):689–703. 10.1515/jpm-2019-0214. 31393835 10.1515/jpm-2019-0214

[13] Li J, Yin J, Waqas A, Huang Z, Zhang H, Chen M, et al. Quality of life in mothers with perinatal depression: A systematic review and meta-analysis. Front Psychiatry. 2022;13:734836. 10.3389/fpsyt.2022.734836. 35242060 10.3389/fpsyt.2022.734836 PMC8886107

[14] Brekke M, Amro A, Småstuen MC, Glavin K, Solberg B, Utne Øygarden AM, et al. Quality of life in Norwegian pregnant women and men with pregnant partners, and association with perception of sleep and depressive symptoms: A cross-sectional study. BMC Pregnancy Childb. 2023;23(1):37. 10.1186/s12884-023-05379-x. 10.1186/s12884-023-05379-x PMC9847178 36653752

[15] Delale EA, Novokmet N, Fuchs N, Dolanc I, Mrdjen-Hodžić R, Karelović D, et al. Stress, locus of control, hope and depression as determinants of quality of life of pregnant women: Croatian islands’ birth cohort study (CRIBS). Health Care Women Int. 2021;42(12):1358–78. 10.1080/07399332.2021.1882464. 33900158 10.1080/07399332.2021.1882464

[16] Bae BR. Structural equation modeling with AMOS 27. Seoul: Chungram Publishing; 2021.

[17] Min SK, Kim KI, Lee CI, Jung YC, Suh SY, Kim DK. Development of the Korean versions of WHO quality of life scale and WHOQOL-BREF. Qual Life Res. 2002;11(6):593–600. 10.1023/a:1016351406336. 12206580 10.1023/a:1016351406336

[18] Sohn SI, Kim DH, Lee MY, Cho YW. The reliability and validity of the Korean version of the Pittsburgh sleep quality index. Sleep Breath. 2012;16(3):803–12. 10.1007/s11325-011-0579-9. 21901299 10.1007/s11325-011-0579-9

[19] Buysse DJ, Reynolds CF, Monk TH, Berman SR, Kupfer DJ. The Pittsburgh sleep quality index: A new instrument for psychiatric practice and research. Psychiatry Res. 1989;28:193–213. 10.1016/0165-1781(89)90047-4. 2748771 10.1016/0165-1781(89)90047-4

[20] Chon KK, Choi SC, Yang BC. Integrated adaptation of CES-D in Korea. Korean J Health Psychol. 2001;6(1):59–76. 10.4236/ojmp.2012.14009.

[21] Liang CC, Chao M, Chang SD, Chiu SYH. Impact of pre-pregnancy body mass index on pregnancy outcomes, incidence of urinary incontinence and quality of life during pregnancy - An observational cohort study. BMJ. 2020;43(6):476–83. 10.1016/j.bj.2019.11.001. 10.1016/j.bj.2019.11.001 PMC7804172 33246799

[22] Amador-Licona N, Guízar-Mendoza JM. Daytime sleepiness and quality of life: are they associated in obese pregnant women? Arch Gynecol Obstet. 2012;285(1):105–9. 10.1007/s00404-011-1879-9. 21437629 10.1007/s00404-011-1879-9

[23] Louis J, Auckley D, Miladinovic B, Shepherd A, Mencin P, Kumar D, et al. Perinatal outcomes associated with obstructive sleep apnea in obese pregnant women. Obstet Gynecol. 2012;120(5):1085–92. 10.1097/AOG.0b013e31826eb9d8. 23090526 10.1097/AOG.0b013e31826eb9d8 PMC3552141

[24] Mohammadi M, Maroufizadeh S, Omani-Samani R, Almasi-Hashiani A, Amini P. The effect of prepregnancy body mass index on birth weight, preterm birth, Cesarean section, and preeclampsia in pregnant women. J Matern Fetal Neonatal Med. 2019;32(22):3818–23. 10.1080/14767058.2018.1473366. 29768986 10.1080/14767058.2018.1473366

[25] Austin MP, Middleton P, Reilly NM, Highet NJ. Detection and management of mood disorders in the maternity setting: the Australian clinical practice guidelines. Women Birth. 2013;26(1):2–9. 10.1016/j.wombi.2011.12.001. 22217978 10.1016/j.wombi.2011.12.001

[26] Jimènez-Barragan M, Falguera-Puig G, Curto-Garcia JJ, Monistrol O, Coll-Navarro E, Tarragó-Grima M, et al. Prevalence of anxiety and depression and their associated risk factors throughout pregnancy and postpartum: A prospective cross-sectional descriptive multicentred study. BMC Pregnancy Childb. 2024;24(1):500. 10.1186/s12884-024-06695-6. 10.1186/s12884-024-06695-6 PMC11270936 39054429

[27] Dai H, Mei Z, An A, Wu J. Association between sleep problems and health-related quality of life in Canadian adults with chronic diseases. Sleep Med. 2019;61:26–30. 10.1016/j.sleep.2019.04.015. 31255481 10.1016/j.sleep.2019.04.015

[28] Zhang J, Chai X, Ye Y, Zhao Q, Fan X. Association between sleep and quality of life in heart failure patient-caregiver dyads and mediation of fatigue: an actor-partner interdependence mediation model. J Adv Nurs. 2022;78(8):2436–47. 10.1111/jan.15174. 35133026 10.1111/jan.15174

[29] Zhi TF, Sun XM, Li SJ, Wang QS, Cai J, Li LZ, et al. Associations of sleep duration and sleep quality with life satisfaction in elderly chinese: the mediating role of depression. Arch Gerontol Geriatr. 2016;65:211–7. 10.1016/j.archger.2016.03.023. 27100684 10.1016/j.archger.2016.03.023

[30] Theriault CB, DiPlacido J, Zempsky WT, Santos M. The relationship between experiences of pain among youth with obesity and health-related quality of life: the role of functional limitation, sleep, and depressive symptoms. Child Obes. 2024;20(2):87–95. 10.1089/chi.2022.0203. 36877538 10.1089/chi.2022.0203

[31] Woo D, Lee Y, Park S. Associations among working hours, sleep duration, self-rated health, and health-related quality of life in Korean men. Health Qual Life Outcomes. 2020;24(1):287. 10.1186/s12955-020-01538-2. 10.1186/s12955-020-01538-2 PMC7444202 32831092

[32] Gu R, Chen H, Wang X, Jin X, Jiang F, Zhao W, et al. The mediating role of appraisal on health-related quality of life in adolescent and young adult cancer survivors. Qual Life Res. 2023;32(4):1069–84. 10.1007/s11136-022-03269-x. 36260164 10.1007/s11136-022-03269-x

[33] Lee SY, Hsu HC. Stress and health-related well-being among mothers with a low birth weight infant: the role of sleep. Soc Sci Med. 2012;74(7):958–65. 10.1016/j.socscimed.2011.12.030. 22342365 10.1016/j.socscimed.2011.12.030 PMC3464912

[34] Xu J, Ji Q, Ji P, Chen Y, Song M, Ma J, et al. The relationship between sleep quality and quality of life in middle-aged and older inpatients with chronic diseases: mediating role of frailty and moderating role of self-esteem. Geriatr Nurs. 2025;61:681–8. 10.1016/j.gerinurse.2024.10.051. 39516094 10.1016/j.gerinurse.2024.10.051

[35] Banerjee S, Boro B. Analyzing the role of sleep quality, functional limitation and depressive symptoms in determining life satisfaction among the older population in india: a moderated mediation approach. BMC Public Health. 2022;18(1):1933. 10.1186/s12889-022-14329-9. 10.1186/s12889-022-14329-9 PMC9578239 36258170

[36] Yuan Y, Li J, Jing Z, Yu C, Zhao D, Hao W, Zhou C. The role of mental health and physical activity in the association between sleep quality and quality of life among rural elderly in china: A moderated mediation model. J Affect Disord. 2020;273:462–7. 10.1016/j.jad.2020.05.093. 32560941 10.1016/j.jad.2020.05.093

[37] Korean Statistical Information Service. https://kosis.kr/index/index.do. Accessed on 3 Nov 2025.

